# Effects of Five Amino Acids (Serine, Alanine, Glutamate, Aspartate, and Tyrosine) on Mental Health in Healthy Office Workers: A Randomized, Double-Blind, Placebo-Controlled Exploratory Trial

**DOI:** 10.3390/nu14112357

**Published:** 2022-06-06

**Authors:** Kentaro Umeda, Daichi Shindo, Shinji Somekawa, Shinobu Nishitani, Wataru Sato, Sakiko Toyoda, Sachise Karakawa, Mika Kawasaki, Tomoyuki Mine, Katsuya Suzuki

**Affiliations:** 1 Institute of Food Sciences and Technologies, Ajinomoto Co., Inc., Kawasaki 210-8681, Japan; daichi.shindo.km5@asv.ajinomoto.com (D.S.); shinji.somekawa.mr9@asv.ajinomoto.com (S.S.); shinobu.nishitani.vx8@asv.ajinomoto.com (S.N.); 2 Research Institute for Bioscience Products & Fine Chemicals, Ajinomoto Co., Inc., Kawasaki 210-8681, Japan; wataru.sato.m7h@asv.ajinomoto.com (W.S.); sakiko.toyoda.r4s@asv.ajinomoto.com (S.T.); sachise.karakawa.tn3@asv.ajinomoto.com (S.K.); mika.kawasaki.2k5@asv.ajinomoto.com (M.K.); 3 Research & Business Planning Department, Ajinomoto Co., Inc., Tokyo 104-8315, Japan; tomoyuki.mine.65v@asv.ajinomoto.com

**Keywords:** mental health, amino acids, motivation, cognitive function

## Abstract

Background: The importance of maintaining good mental health with overall well-being has recently drawn attention from various spheres of academics and the working population. Amino acid intake has been reported to reduce depression symptoms and other mental health problems. However, the effectiveness of amino acid intake (i.e., single or combined) remains unknown. In this study, we assessed a combination of five amino acids (serine, alanine, glutamate, aspartate, and tyrosine; SAGAT) reported to regulate mental health. Methods: A randomized, double-blind, placebo-controlled exploratory trial was conducted. Participants, aged between 20 and 65 years with fatigue sensation, were randomized to receive either SAGAT or the placebo and ingested them for four weeks. A transient mental work was loaded at day 0 and after four weeks of intervention. As the primary outcomes, the fatigue sensation was assessed. The mood status, cognitive function, work efficiency, and blood marker were also measured as secondary outcomes. Results: The number of participants analyzed for the efficacy evaluation were 20 in SAGAT and 22 in the placebo. There were no significant differences in the primary outcomes. However, as the secondary outcomes, the SAGAT group showed a significant improvement in motivation and cognitive function in the recovery period after mental work loaded in a four-week intervention compared to the placebo. Conclusion: The current findings suggest that SAGAT contributes to maintaining proper motivation and cognitive function. Clinical Trial Registration: University Hospital Medical Information Network Clinical Trial Registry (ID: UMIN 000041221).

## 1. Introduction

In modern society, reducing stress is a major issue for improving work engagement among workers. The number of patients with psychiatric disorders associated with working, such as depression, has increased since 2000 in Japan, and there has been a strong need to develop solutions to workers’ stress and fatigue [1]. Furthermore, the recent COVID-19 pandemic has caused public panic and mental health problems in various sectors of the global population. A systematic review and meta-analysis showed that the global prevalence estimate was 28.0% for depression, 26.9% for anxiety, 24.1% for post-traumatic stress symptoms, 36.5% for stress, 50.0% for psychological distress, and 27.6% for sleep problems [2]. A review indicated that anxiety, depression, and distress increased in the early months of the pandemic, and ways to support mental health have been recommended [3]. These include exercise, nutrition, and diet, which are important lifestyle factors for mental health [4].

Recent studies suggest that one of the causes of fatigue is stress-induced peripheral and central inflammation. In a mouse model exposed to mental stress, when inflammatory cytokines in the blood and brain were increased, cognitive function decreased [5]. Another study on patients with chronic fatigue syndrome who experienced increased inflammation in the brain found a correlation between inflammation in the thalamus and cognitive decline [6]. Furthermore, mental disorders, such as depression and anxiety, are frequently caused by an imbalance of neurotransmitters in the brain. Depression is reported to be associated with an imbalance of serotonin, norepinephrine, and dopamine, and reduced levels of γ-aminobutyric acid (GABA) lead to anxiety and mood disorders [7]. The amino acids that are the precursors of these neurotransmitters and their metabolites, such as phenylalanine, tyrosine, tryptophan, and kynurenine, are also reported to be associated with mental stress. Specifically, acute mental stress has been reported to upregulate kynurenine/tryptophan in healthy students [8]. Moreover, the maintenance of neural conditions is important for the regulation of mental health. There is a relationship between neuronal axonal damage and fatigue in multiple sclerosis [9]. These reports suggest that several factors, including inflammation and abnormalities in neurotransmitters and amino acid metabolites, affect psychoneurotic abnormalities such as fatigue and mental disorders.

Growing evidence suggests that certain food ingredients affect fatigue recovery, mood improvement, and stress reduction. For example, the daily intake of chicken essence has been reported to aid the recovery from mental fatigue in healthy men [10], and the oral administration of GABA has been known to help reduce stress and fatigue [11,12,13,14]. Recent studies on the gut microbiome suggest that probiotic materials are important solutions for not only the intestinal environment but, also, mood conditions [15,16].

Supplements containing amino acids have also been found to reduce symptoms of depression and other mental health problems; the administered amino acids are converted into neurotransmitters that alleviate mental problems [17]. Tyrosine (Tyr) is a neurotransmitter precursor known to reduce environmental stress in humans [18]. In patients with primary biliary cirrhosis, the concentration of plasma Tyr has been associated with fatigue and a diminished quality of life [19]. A systematic review concluded that Tyr intake can have beneficial effects on psychological functioning in healthy human adults [20]. Serine (Ser) is important for maintaining the normal function of the nervous system by acting as a precursor for sphingolipids and glycolipids, which are important membrane components and myelin constituents [21]. Recently, the combination of Ser and eicosapentaenoic acid has been reported to be effective for pain relief in adults with low back and knee pain mediated by their neuroprotective function [22]. The ingestion of glutamate (Glu) during the developmental period reduced aggressive behavior in a model rat with an attention-deficit hyperactivity disorder, and this effect is mediated by gut–brain interaction [23]. Alanine (Ala) has been reported to modulate anxiety-like behavior in rats [24], and aspartate (Asp) is known to increase the contribution of oxidative metabolism in energy production and delay fatigue during exercise [25].

Based on the above, we hypothesized that the combined intake of these amino acids that might affect neurotransmitters, their precursors, and metabolites would have a beneficial effect on the regulation of mental health. In this study, we evaluated the combinational effects of five amino acids (Ser, Ala, Glu, Asp, Tyr; SAGAT) on the mental health of office workers. The results highlighted the potential effects of four weeks of SAGAT ingestion on the motivation and cognitive function in the recovery phase after the transient mental work was loaded. This was the first study to show the combinational effects of SAGAT, even though each amino acid has been reported to improve mental health problems or cognitive functions.

## 2. Materials and Methods

### 2.1. Study Design

**Figure 1:**
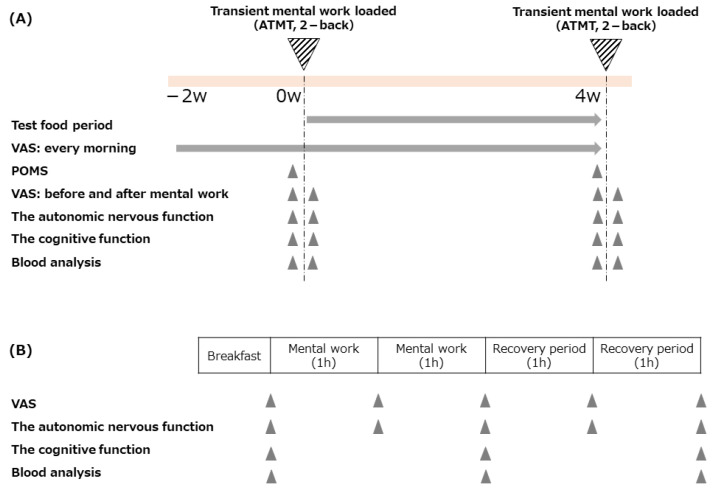
The outline of this study. (**A**) The overall outline of the test period. (**B**) The schedule details for days with transient mental work loaded.

A randomized, double-blind, placebo-controlled trial was conducted between August and November 2020 in Osaka, Japan. The study is outlined in Figure 1. The visual analogue scale (VAS) for fatigue [26], face scale [27], Chalder fatigue scale [28], and profile of mood states questionnaire second edition (POMS2) Japanese version (Kaneko Shobo Co., Ltd., Tokyo, Japan) were used at the point of screening. Autonomic nerve function was evaluated using the fatigue measurement device VM302 (Hitachi Systems Co. Ltd., Tokyo, Japan), and a cognitive function test using Cog Evo Ri (Total Brain Care Co. Ltd., Kobe, Japan) was also conducted. Physicians performed face-to-face interviews and blood tests.

In the test period, transient mental work was loaded at day 0 and after four weeks of intervention. The transient mental work consisted of two sets (2 h) of 30-min 2-back tasks (simple working memory tasks) [29,30] and 30 min of the advanced trail making test (ATMT), a selective attention and spatial working memory task [29,31]. The VAS scores for fatigue, relaxation, tension, motivation, concentration, and sleepiness were recorded in various situations during the test period, as shown in Figure 1. The effects of the test food intake on the daily mood status were verified using POMS2 at day 0 and after four weeks of intervention. Autonomic and cognitive functions were recorded both before and after transient mental work that was asked to be performed on day 0 and after four weeks of intervention. The results of transient mental work (2-back task and ATMT) are discussed as the participants’ work efficiency.

The biological blood tests and the medical doctors’ face-to-face interviews were performed both before and after transient mental work were asked to be performed at day 0 and after four weeks of intervention. This study was conducted in compliance with the Declaration of Helsinki and with the approval of the Institutional Review Board of Fukuda Clinic (approval number: IRB-20200718-4) and the Ethics Committee of Ajinomoto Co., Inc. (approval code: 2019-017). The study was registered in the University Hospital Medical Information Network Clinical Trial Registry (https://center6.umin.ac.jp/cgi-open-bin/ctr_e/ctr_view.cgi?recptno=R000047073, accessed on 14 April 2022, ID: UMIN 000041221).

### 2.2. Participants

**Figure 2:**
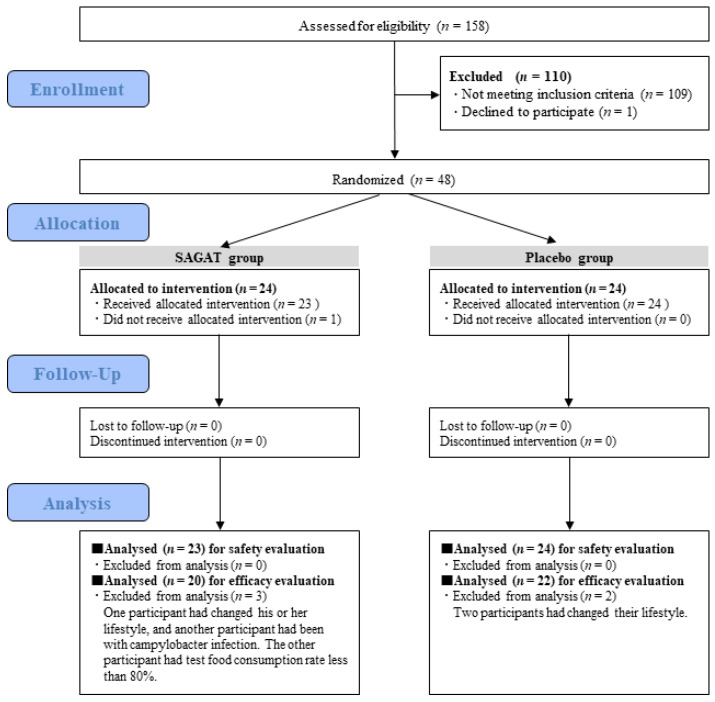
The study flow diagram.

Participants in this study were healthy Japanese men and women aged between 20 and 65 years and office workers at the time of consent. The eligibility criteria for the inclusion of the participants were as follows: (1) those who answered the questionnaire that they had fatigue sensation during daily life; (2) those who were deemed to be fatigued based on their VAS and face scale scores at the screening; (3) those who worked in an office; and (4) those who received a sufficient briefing of the objective and content of the present study, fully understood and agreed to voluntarily participate in this study, and were able to sign a written informed consent. The exclusion criteria were as follows: (1) those who received medical treatment for serious cardiovascular, hepatic, renal, respiratory, endocrine, or metabolic disorders or had a medical history of these disorders; (2) those who had chronic fatigue syndrome or were deemed by the investigator to have severe fatigue such as idiopathic chronic fatigue; (3) those who took medicines or quasi-drugs regularly that enabled recovery from fatigue or took nutritional supplements during physical fatigue; (4) those who regularly took Foods with Function Claims, the labels of which mentioned the effect of attenuating fatigue sensation; (5) those who regularly took supplements of amino acids or proteins; (6) those who were heavy drinkers (more than 60 g of pure alcohol per day); (7) those with a body mass index (BMI) of less than 18.5 or 30 or more; (8) those who had the possibility of primary diseases in the autonomic nervous system; (9) those who had a blood sample of 200 mL or more taken within one month or 400 mL or more within three months prior to the start of the study (e.g., donated blood); (10) those who took part in another clinical study within three months prior to the start of the present study or were taking part in another clinical study; (11) women who were pregnant or lactating or intended to become pregnant during the test period; and (12) those who were deemed unsuitable by the investigator. Since the present study was conducted as an exploratory study, the sample size was not calculated; however, the number of participants in this study was determined based on previous reports investigating the effect of food ingredients on mental health, such as reducing stress [32,33]. A total of 158 participants were screened, and 48 were included and randomly assigned to two groups using stratified randomization allocation based on their ages, gender, BMI, VAS (fatigue), Chalder fatigue scale, LF/HF, TP, and high-sensitivity C-reactive protein (CRP) by a staff member of Statcom Co., Ltd. (Tokyo, Japan) who did not directly participate in this trial. Allocation to each test food group was concealed from participants, investigators, technicians, data analysts, evaluators, and the medical doctor until the study was completed. Six individuals were excluded from the analysis for the following reasons: three changed their lifestyles during the intervention, and one had Campylobacter infection. One had chyle serum, and another had a test food consumption rate of less than 80%. Figure 2 shows the study flow diagram and the number of participants in each group.

### 2.3. Test Food

The granule powder containing five amino acids (0.50 g L-Ser, 1.00 g D,L-Ala, 1.00 g L-Asp (sodium L-Asp), 0.50 g L-Glu, and 1.00 g L-Tyr) was put in a sachet. The placebo-controlled food contained cellulose and isomaltulose instead of amino acids. We verified that there was no difference in flavor or appearance between the test food and the placebo-controlled food. The participants’ daily intake was recorded.

### 2.4. Outcomes

The primary outcomes of this study were VAS for fatigue (the parameter of the subjective fatigue sensation) and autonomic nervous function (ANF; the parameter of the objective fatigue sensation). The secondary outcomes were mood status, work efficiency, cognitive function, and blood analysis.

#### 2.4.1. Primary Outcomes

##### Fatigue Sensation

The subjective fatigue sensation was assessed using the VAS, which is recommended in the guidelines of the Japanese Society of Fatigue Science [34]. To assess daily mental health, VAS scores for fatigue were recorded within 30 min after waking up and before breakfast on weekdays from two weeks before the intervention to the end of four weeks of intervention. As for the assessment of transient work load mental health, VAS scores for fatigue were recorded before and after the transient workload experienced by the participants at day 0 and after four weeks of intervention. The score was presented on a scale ranging from 0 to 100, with a higher score representing a higher level of fatigue feeling.

##### Autonomic Nervous Function

The objective fatigue sensation was assessed by ANF recorded using the fatigue measurement device VM302 before and after transient work was loaded on days 0 and after four weeks of intervention. The VM302 evaluates ANF by monitoring HRV in 90-s electrocardiogram interval data, which are transmitted to an external computer [35].

#### 2.4.2. Secondary Outcomes

##### Mood Status

Subjective daily mood sensations, such as motivation, sleepiness, and exhilaration, were recorded using VAS within 30 min after waking up and before breakfast on weekdays from two weeks before intervention to the end of four weeks of intervention. In addition, regarding transient mental work-loaded mental health, VAS scores for relaxation, tension, motivation, concentration, and sleepiness were recorded before and after the transient workload experienced by the participants at day 0 and after four weeks of intervention. The daily mood status was also evaluated using the POMS2 at two points (before and after intervention). The reliability and validity of POMS2 have been confirmed, and it is widely used in medical and industrial fields [36]. Sixty-five questions in this self-reported measurement instrument were classified into seven mood subscales: (1) Anger/Hostility, (2) Confusion/Bewilderment, (3) Depression/Dejection, (4) Fatigue/Inertia, (5) Tension/Anxiety, (6) Vigor/Activity, and (7) Friendliness. The other questionnaire for fatigue using the Chalder fatigue scale [28] was recorded at three points (before intervention and at two and at four weeks of intervention).

##### Cognitive Function

Cognitive function was assessed using Cog Evo Ri, a computer-aided neuropsychiatric series of tests, which can be used to evaluate the age-related or pathological decline in cognitive function from middle age and in preclinical stages of dementia [37] before and after the transient work was loaded at day 0 and after four weeks of intervention.

##### Blood Marker

To assess the effects on mental health-related changes, high-sensitivity CRP, amino acid metabolite (tryptophan and kynurenine) [38], oxidation markers (d-ROMs and BAP), and antioxidant markers (uric acid) were measured before and after transient work at day 0 and after four weeks of intervention. To evaluate food safety, a hematological examination was performed before and after four weeks of intervention.

##### Work Efficiency

Transient mental work, 2-back task, and ATMT were performed before and after four weeks of intervention. The results of the (ABC) task were assessed as the work efficiency [31].

### 2.5. Statistics Analysis

All results were expressed as the mean ± standard deviation (SD). All analyses were performed using GraphPad Prism, Ver. 6.0 (GraphPad Software Inc., San Diego, CA, USA), and Microsoft Excel 2013 (Microsoft Japan Co., Ltd., Tokyo, Japan) was also used. Results were considered to be statistically significant at *p* < 0.05.

#### 2.5.1. Analysis of Daily VAS

Regarding the intergroup comparisons, an unpaired *t*-test was used when the data were homoscedastic, while Welch’s test was used to analyze the data when homoscedasticity could not be assumed. For the pre- and post-intervention comparisons, the results were analyzed using repeated measures ANOVA, followed by Dunnett’s multiple comparison test.

#### 2.5.2. Analysis of Transient Work Loaded-VAS, Autonomic Nervous Function, Cognitive Function, Blood Markers

When the mental work was not loaded, an unpaired *t*-test was used for intergroup comparisons when data were homoscedastic, and Welch’s test was used to analyze data when homoscedasticity could not be assumed. For the pre- and post-intervention comparisons, the results were analyzed using a paired *t*-test.

When the mental work was loaded, an unpaired *t*-test and Welch’s test were performed as above. For the pre- and post-intervention comparisons, the results were analyzed using repeated measures ANOVA, followed by Dunnett’s multiple comparison test.

#### 2.5.3. Analysis of Questionnaires (Chalder Fatigue Scale and POMS2)

For the intergroup comparisons of the Chalder fatigue scale, an unpaired *t*-test was used when the data were homoscedastic, while Welch’s test was used to analyze the data when homoscedasticity could not be assumed. For the pre- and post-intervention comparisons of the Chalder fatigue scale, the results were analyzed using repeated measures ANOVA, followed by Dunnett’s multiple comparison test. For the intergroup comparisons of the POMS2 scores, the Mann–Whitney *U* test was used. As for the pre- and post-intervention comparisons of the POMS2 scores, the results were analyzed using the Wilcoxon signed rank test.

#### 2.5.4. Analysis of Work Efficiency (ATMT and 2-Back Task)

The Mann–Whitney *U* test was used for intergroup comparisons, except for the number of errors. Other indices were analyzed by the unpaired *t*-test when the data were homoscedastic. Welch’s test was used to analyze the data when homoscedasticity could not be assumed.

## 3. Results

### 3.1. Participants

The backgrounds of the participants are shown in Table 1. The number of participants for the efficacy evaluation were 20 in SAGAT and 22 in the placebo, and those for safety evaluation were 23 in SAGAT and 24 in the placebo. There were no significant differences observed in age, sex, BMI, VAS (fatigue), LF/HF (ANF), and high-sensitivity CRP.

### 3.2. Primary Outcomes

#### 3.2.1. Fatigue Sensation

The weekly average values of VAS for fatigue recorded every morning during the weekday are shown in Table 2A,B. No significant differences were observed in any timing between the SAGAT and the placebo control groups. As for the assessment of VAS for fatigue when transient work was loaded, the scores recorded before and after transient work loaded at four weeks of intervention are shown in Table 2C,D. After four weeks of intervention, there were no significant differences between the SAGAT and placebo control groups in the observed value of fatigue VAS.

#### 3.2.2. Autonomic Nervous Function

The observed values of LF, HF, and LF/HF before and after the intervention are shown in Table 3A. There were no significant differences between the two groups. As for the assessment of transient work loaded-ANF, the observed values of LF, HF, and LF/HF measured before and after the transient work loaded at four weeks of intervention are shown in Table 3B,C. After four weeks of intervention, there were no significant differences in the observed values between the two groups (Table 3B). As for the changes in the recovery period from before loading mental work in the four weeks of intervention, the SAGAT group had significantly lower LF than the placebo control group (Table 3C). The indicators related to ANF other than LF and HF are shown in Supplemental Table S1.

### 3.3. Secondary Outcomes

#### 3.3.1. Mood Status

Scores for subjective daily mood sensations, such as motivation, sleepiness, and exhilaration using VAS, are shown in Table 4A,B. No significant differences were observed in any of the items between the SAGAT and the placebo control groups. On the other hand, as for the assessment of the transient workload, the subjective mood scores, such as relaxation, tension, motivation, concentration, and sleepiness using VAS, are shown in Table 4C,D. In the amount of change in the motivation score during the recovery period from before performing mental work after four weeks of intervention, the SAGAT group showed a significant improvement compared to the placebo control.

The results of the T-scores for the Japanese version of the POMS2 recorded before and after the intervention are shown in Supplemental Table S2. For all mood indices, there were no significant differences between the two groups. However, regarding the comparison before and after the intervention, only the SAGAT group showed a significant improvement in Confusion–Bewilderment, Depression–Dejection, Fatigue-–Inertia, Tension–Anxiety, and Vigor–Activity scores. The total mood disturbance was also significantly improved after SAGAT intervention.

The scores of other questionnaires for fatigue, the chalder fatigue scale, recorded before at two and at four weeks of the intervention are shown in Supplemental Table S3. No significant differences were observed between the two groups.

#### 3.3.2. Cognitive Function

The cognitive functions scores recorded before and after the intervention are shown in Table 5A. In the observed values, the SAGAT group showed a significantly lower Attention, Stroop test score before intervention, and a significantly lower spatial cognition and Just Fit score after four weeks of intervention compared to the placebo control group. The cognitive functions scores recorded before and after transient work performed at four weeks of the intervention are shown in Table 5B,C. After four weeks of the intervention, the SAGAT group showed a significantly higher orientation and time management score than the placebo control group in the recovery period after the mental work was performed. As for the amount of change in recovery period from before loading mental work in the four-week intervention, the SAGAT group showed significantly higher spatial cognition and Just Fit scores than the placebo control group.

#### 3.3.3. Blood Marker

The results of high-sensitivity CRP, amino acid metabolite (tryptophan and kynurenine), oxidation markers (d-ROMs and BAP), and antioxidant markers (uric acid) measured before and after intervention are shown in Supplemental Table S4A. The placebo control group showed significantly high uric acid after four weeks of intervention. The results of the high-sensitivity CRP and amino acid metabolite oxidation markers measured before and after the transient work performed at four weeks of intervention are shown in Supplemental Table S4B,C. After four weeks of intervention, BAP was found to be significantly lower in the SAGAT group than in the placebo control group 2 h after the mental work was loaded. Furthermore, tryptophan (Trp) was significantly higher in the SAGAT group than in the placebo control group during the recovery period after mental work was loaded.

#### 3.3.4. Work Efficiency

The results of ABC task recorded before and after the intervention are shown in Supplemental Table S5. After four weeks of intervention, the SAGAT group had significantly fewer blank-push-type errors than the placebo control group in the second set. The result of the 2-back task was also assessed as work efficiency, but there were no significant differences between the groups (data not shown).

#### 3.3.5. Evaluation of Test Food Safety

The results of the hematological examinations are shown in Supplemental Table S6. After four weeks of intervention, there were no significant differences in any of the items between the two groups. The physician determined that there were no adverse events associated with SAGAT intake. On the contrary, three participants in the placebo control group had high uric acid values (8.2, 8.6, and 9.5 mg/dL, respectively). The physician considered that this was probably related to sodium inosinate administered to the placebo control group. In the follow-up survey, up to 40 days after the end of the intervention, there were no symptoms that may have occurred with an increase in the uric acid levels.

## 4. Discussion

The present study was a randomized, double-blind, placebo-controlled exploratory trial to investigate the effects of SAGAT intake for four weeks on mental health in healthy office workers aged between 20 and 65 years. Each amino acid contained in SAGAT has been reported to improve mental health problems or cognitive functions. We hypothesized that Tyr and Ser, which directly affect neurons [18,21], function as the main effects of SAGAT, and the other three amino acids promote their effects, because they have been reported to reduce fatigue or mental health-related behavior in animal model studies [23,24,25]. However, there was no research showing the combinational effects of these amino acids.

As for the primary outcomes, there were no significant differences between the SAGAT and placebo control groups in the subjective fatigue sensation assessed by VAS. This may be because, in general, the subjective evaluation is likely to change before and after intervention, even in the control group, in the parallel group comparison test, so that the differences between SAGAT and the placebo could not be detected. The amount of change in fatigue VAS in the placebo group at two weeks of intervention in this study (Table 2) was largely compared to that in a similar study conducted with a crossover design [39].

We also measured the objective fatigue sensation assessed by ANF recorded using the fatigue measurement device VM302. LF/HF, a relative indicator of sympathetic nerve activity, has been reported to be increased during fatigue [40]. However, there were no significant differences in LF/HF before and after the mental workload (Table 3) in either the placebo or SAGAT group. Although there were significant changes in the VAS, subjective fatigue score, after the mental workload (Table 2) in this study, the degree of transient mental work might be insufficient to change the LF/HF ratio. Thus, we cannot discuss the effect of the SAGAT intake on the objective fatigue index.

As for the secondary outcomes, the results showed that a four-week intake of SAGAT regulated motivation and cognitive function in the recovery period after the transient mental work was loaded (Table 4C,D and Table 5B,C). Since this study is an exploratory research, we discuss various possible mechanisms underlying the effectiveness of SAGAT on motivation and cognitive function.

First, one amino acid in SAGAT may especially affect the cognitive function. Tyr has been reported to improve the performance on cognitive tasks in healthy young participants [41], and its beneficial effects on cognitive function are widely attributed to its role as a precursor for the synthesis of norepinephrine and dopamine [42]. These reports suggest that the continuous intake of Tyr for four weeks led to an increase in neurotransmitter precursors and the regulation of cognitive function.

Second, SAGAT might be effective in improving nerve damage caused by transient mental work loading. We recruited participants who confirmed that they faced fatigue in daily life and who were deemed to be fatigued based on their scores on the VAS and face scale at the screening. Therefore, the participants in this study had relatively high fatigue rates or poor mental health. In certain conditions, such as multiple sclerosis, a relationship exists between neuronal axonal damage and fatigue [9]. Although we did not measure the conditions of neurons in the participants, it is possible that the participants in this study had some nerve damage due to transient mental work. Ser is important for maintaining normal function of the nervous system by acting as a precursor for sphingolipids and glycolipids, which are important membrane components and myelin constituents [21]; the intake of Ser for four weeks may have made neurons resistant to transient stress-induced damage and maintain proper nerve conditions.

Third, previous reports showed that the ingestion of monosodium glutamate (MSG) indirectly induced behavioral changes through gut–brain interaction mediated by the vagus nerve in a rat with an attention deficit hyperactivity disorder [23], suggesting that the oral intake of Glu caused the interaction of the gut–brain axis. Furthermore, MSG supplementation is known to increase gastric acid secretion in patients with chronic gastritis [43], and the umami taste is the most potent taste stimulus for saliva secretion from the parotid gland [44]. These reports suggested that the intake of Glu enhanced a proper appetite and enriched daily diets, resulting in the improvement of mood status.

Fourth, two amino acids in SAGAT (Asp and Ala) may contribute to improving mental health in terms of energy repair. Abnormal energy metabolism has been reported in fatigued rats and patients with chronic fatigue syndrome [45,46]. Ala and Asp are known to be involved in gluconeogenesis and serve as the main energy sources in the body [47,48]. Therefore, a continuous four-week intake of Ala and Asp may maintain normal energy metabolism and have positive effects on energy repair after transient mental work is loaded.

Fifth, each effect of SAGAT on motivation and cognitive function may has been enhanced by crosstalk among them. The self-reports of intrinsic motivation were reported to be strongly related to cognitive performance [49]. In our data, the change in motivation VAS score was improved significantly in the SAGAT group compared to placebo 1 h after recovery, and this was also observed 2 h after recovery (Table 4). In addition, the scores of the cognitive functions 2 h after recovery were significantly improved in SAGAT compared to the placebo (Table 5). These results suggested the improvement of motivation in early stage of the recovery period had a positive effect on the cognitive function in late phase of recovery period. However, it is unclear why the SAGAT intake was effective only on a specific cognitive function (i.e., Spatial cognition, Just Fit), and further research will be required to address this issue.

Regarding blood markers, we measured high-sensitivity CRP, amino acid metabolites, d-ROMs, and BAP as fatigue has been reported to be associated with inflammation and oxidative stress [6,50]. However, the present study showed no significant differences in blood markers between the groups except for uric acid. Although ATMT has been reported to cause mental fatigue [31], it is possible that this mental workload was not sufficient to cause inflammation, changes in amino acid metabolites, and oxidation markers among participants in the current study. At the point of screening, those who participated in this study showed a relatively high degree of fatigue, but the value of high-sensitivity CRP before intervention was found to be lower than in other reports that employed patients who faced cancer-related fatigue and had chronic fatigue syndrome [51]. Further studies will be required to set recruitment criteria by combining fatigue VAS with the values of fatigue-related markers of the blood.

This study has some limitations. It was an exploratory study, and the generalizability of the obtained findings is limited. First, because of the small sample size of the present study, additional studies with an appropriate sample size, calculated based on the results of this study, are required. Second, we used per protocol set data for the analysis of the effect of SAGAT. The present study was conducted as an exploratory pilot trial to evaluate the potential effect of the combination of SAGAT on mental health. Therefore, we excluded the participants who did not meet the analysis criteria to make it easier to observe the effect of SAGAT intake. Third, the blood uric acid levels were altered in the placebo control group of this study. The physician considered that this was probably related to sodium inosinate administered to the participants of the placebo group, and it cannot be completely ruled out that this change did not affect the evaluation of the effects of the test food on mental health. However, the association between blood uric acid and mental health-related disorders, such as depression and anxiety, has not been clearly shown [52,53] and needs further discussion.

## 5. Conclusions

In conclusion, the data of this study highlight the potential effects of intake of five amino acids (SAGAT) for four weeks on the motivation and cognitive function in the recovery period after transient mental work is performed. Our findings suggest that taking a combination of amino acids that are known to be effective for mental health has the potential to contribute to the maintaining proper motivation and cognitive function in office workers. Future studies will be needed to clarify the action mechanisms of SAGAT on mental health using participants who are recruited based on items for which the usefulness of SAGAT was confirmed in this study.

## Figures and Tables

**Table 1:** Backgrounds of the participants.

(A) FAS							
Group (*n*)	M	F	age (years)	BMI (kg/m^2^)	VAS (fatigue)	Autonomic nervous function (LF/HF)	High-sensitivity CRP (mg/dL)
SAGAT (23)	9	14	46.0 ± 11.5	22.7 ±2.6	58.6 ± 10.2	2.1 ± 2.5	0.05 ± 0.05
Placebo (24)	11	13	45.5 ± 10.2	22.7 ± 1.9	58.4 ± 9.8	2.1 ± 1.8	0.06 ± 0.07
(B) PPS							
Group (*n*)	M	F	age (years)	BMI (kg/m^2^)	VAS (fatigue)	Autonomic nervous function (LF/HF)	High-sensitivity CRP (mg/dL)
SAGAT (20)	8	12	48.0 ± 10.4	22.9 ± 2.6	57.9 ± 10.6	2.3 ± 2.6	0.05 ± 0.05
Placebo (22)	10	12	46.2 ± 10.0	22.6 ± 1.9	59.3 ± 9.4	1.9 ± 1.7	0.05 ± 0.07

The mean and standard deviation (SD) of sex, age, BMI, VAS (fatigue), autonomic nervous function (LF/HF), and high-sensitivity CRP are shown. BMI: body mass index, VAS: visual analogue scale, LF: low frequency, HF: High frequency, CRP: C-reactive protein, (A) FAS: full analysis set, and (B) PPS: per protocol set.

**Table 2:** VAS for fatigue.

(A)					
fatigue	Before	1 week	2 weeks	3 weeks	4 weeks
SAGAT	55.7 ± 11.9	55.4 ± 17.5	47.1 ± 14.3 §§	45.9 ± 16.5 §§	45.2 ± 16.5 §§
Placebo	52.9 ± 12.8	48.7 ± 14.2	45.6 ± 15.1 §§	42.3 ± 17.1 §§	40.9 ± 17.4 §§
(B)					
Δfatigue	1 week	2 weeks	3 weeks	4 weeks	
SAGAT	−0.3 ± 12.2	−8.6 ± 10.2	−9.7 ± 11.4	−10.5 ± 11.4	
Placebo	−4.3 ± 8.5	−7.3 ± 9.9	−10.6 ± 13.2	−12.0 ± 16.3	
(C)					
fatigue	Before Load	1 h after Load	2 h after Load	1 h after Recovery	2 h after Recovery
SAGAT	41.1 ± 17.8	56.5 ± 17.6 §§	59.8 ± 17.1 §§	44.2 ± 14.9	38.5 ± 18.5
Placebo	37.1 ± 16.0	50.9 ± 12.0 §§	56.1 ± 11.7 §§	42.1 ± 16.1	38.2 ± 14.0
(D)					
Δfatigue	1 h after Load	2 h after Load	1 h after Recovery	2 h after Recovery	
SAGAT	15.4 ± 13.3	18.7 ± 13.3	3.1 ± 10.9	−2.7 ± 10.3	
Placebo	13.8 ± 10.7	19.0 ± 10.2	5.0 ± 8.2	1.1 ± 8.7	

(A,C) The mean and SD of each weekly VAS for fatigue, recorded every morning during the weekday (A) and recorded before and after transient work loading at four weeks of intervention (C), are shown. §§ *p* < 0.01 (comparison before and after intervention). (B,D) The mean and SD of changes in each VAS for fatigue from before the intervention (B) and from before mental work loading at four weeks of intervention (D) are shown.

**Table 3:** Autonomic nervous function.

(A)							
		Before	4 weeks	Changes before and after intervention			
LF (ms^2^)	SAGT	439.9 ± 490.7	442.7 ± 402.6	2.8 ± 277.5			
	Placebo	278.6 ± 221.7	510.8 ± 1057.6	253.0 ± 942.7			
HF (ms^2^)	SAGT	414.3 ± 354.9	353.6 ± 326.5	−60.7 ± 260.4			
	Placebo	340.8 ± 377.4	387.9 ± 674.9	37.0 ± 519.8			
LF/HF	SAGT	1.4 ± 1.0	2.1 ± 1.7	0.6 ± 1.8			
	Placebo	1.3 ± 1.3	2.0 ± 2.7	0.8 ± 1.9			
(B)							
		Before Load	1 h after Load	2 h after Load		1 h after Recovery	2 h after Recovery
LF (ms^2^)	SAGAT	442.7 ± 402.6	289.3 ± 200.2	259.9 ± 173.7 §		280.3 ± 213.7	307.6 ± 275.5
	Placebo	510.8 ± 1057.6	687.5 ± 1554.0	405.1 ± 426.2		602.7 ± 915.7	401.6 ± 423.3
HF (ms^2^)	SAGAT	353.6 ± 326.5	296.2 ± 265.1	337.1 ± 345.8		325.5 ± 265.1	481.7 ± 482.2
	Placebo	387.9 ± 674.9	527.8 ± 1172.6	391.5 ± 425.6		562.8 ± 641.8	485.7 ± 508.0
LF/HF	SAGAT	2.1 ± 1.7	2.1 ± 2.6	1.7 ± 1.9		1.8 ± 2.2	1.4 ± 1.9
	Placebo	2.0 ± 2.7	2.8 ± 4.7	2.2 ± 3.7		2.3 ± 2.9	1.4 ± 1.5
(C)							
		1 h after Load	2 h after Load	1 h after Recovery		2 h after Recovery	
LF (ms^2^)	SAGAT	−153.4 ± 417.6	−182.8 ± 304.4	−162.4 ± 319.7	] *	−135.1 ± 450.7	
	Placebo	176.7 ± 638.7	−105.7 ± 719.1	91.9 ± 339.3		−109.3 ± 729.5	
HF (ms^2^)	SAGAT	−57.4 ± 219.0	−16.5 ± 148.4	−28.1 ± 181.8		128.1 ± 353.2	
	Placebo	139.9 ± 563.4	3.5 ± 423.3	174.9 ± 452.9		97.7 ± 403.4	
LF/HF	SAGAT	0.0 ± 3.0	−0.3 ± 2.0	−0.3 ± 2.3		−0.7 ± 2.1	
	Placebo	0.8 ± 4.9	0.2 ± 1.2	0.3 ± 2.0		−0.5 ± 2.5	

(A) The mean and SD of each index for ANF recorded before and after the intervention are shown. (B) The mean and SD of each index for ANF recorded before and after transient work loading at four weeks of intervention are shown. § *p* < 0.05 (comparison before and after mental work loading). (C) The means and SD of changes in each index from before mental work loading at four weeks of intervention are shown. * *p* < 0.05 (comparison between groups). LF: low frequency, HF: High frequency.

**Table 4:** VAS for mood.

(A)								
		Before	1 week	2 weeks		3 weeks		4 weeks
Sleepiness	SAGAT	57.4 ± 14.3	55.5 ± 19.2	48.6 ± 15.5 §		50.0 ± 17.0		46.6 ± 18.1 §§
	Placebo	52.9 ± 13.6	51.1 ± 13.8	47.9 ± 14.5		44.5 ± 16.9 §§		42.9 ± 18.0 §
Motivation	SAGAT	45.1 ± 11.5	45.8 ± 17.8	52.7 ± 14.5 §§		52.4 ± 16.6 §		54.6 ± 17.3 §§
	Placebo	47.7 ± 12.4	50.0 ± 12.5	54.3 ± 14.6 §		57.1 ± 17.0 §		59.8 ± 16.7 §§
Exhilaration	SAGAT	41.2 ± 14.0	44.0 ± 18.9	50.2 ± 16.0 §§		50.6 ± 17.9 §		53.5 ± 18.1 §§
	Placebo	44.3 ± 12.9	48.8 ± 12.2	53.4 ± 15.5 §		57.2 ± 16.9 §§		58.4 ± 18.1 §§
(B)								
		1 week	2 weeks	3 weeks		4 weeks		
ΔSleepiness	SAGAT	−1.9 ± 14.7	−8.8 ± 11.8	−7.4 ± 12.9		−10.8 ± 14.1		
	Placebo	−1.8 ± 9.2	−5.0 ± 10.0	−8.4 ± 10.7		−10.0 ± 14.4		
ΔMotivation	SAGAT	0.7 ± 10.9	7.6 ± 8.1	7.3 ± 10.9		9.5 ± 11.6		
	Placebo	2.3 ± 5.3	6.5 ± 10.5	9.4 ± 14.1		12.1 ± 15.8		
ΔExhilaration	SAGAT	2.8 ± 12.6	9.0 ± 10.6	9.4 ± 12.9		12.3 ± 12.2		
	Placebo	4.6 ± 8.3	9.1 ± 13.2	13.0 ± 14.9		14.2 ± 18.1		
(C)								
		Before Load	1 h after Load	2 h after Load		1 h after Recovery		2 h after Recovery
Tension	SAGAT	40.5 ± 18.7	47.4 ± 17.3 §§	47.1 ± 19.3		40.0 ± 15.0		35.0 ± 16.3
	Placebo	36.7 ± 14.9	42.6 ± 14.5	46.2 ± 12.9 §§		36.9 ± 16.9		33.1 ± 15.5
Sleepiness	SAGAT	44.6 ± 20.7	59.6 ± 17.6 §§	59.4 ± 21.7 §§		44.8 ± 17.0		37.5 ± 19.2
	Placebo	42.8 ± 18.4	55.5 ± 15.1 §§	56.6 ± 17.9 §§		42.0 ± 18.6		41.2 ± 18.7
Relaxation	SAGAT	58.9 ± 18.4	47.5 ± 17.8 §§	50.7 ± 19.7		61.8 ± 13.6		66.2 ± 15.3
	Placebo	62.8 ± 17.3	49.3 ± 11.1 §§	48.0 ± 12.1 §§		62.5 ± 16.6		64.8 ± 16.2
Motivation	SAGAT	55.1 ± 17.0	43.7 ± 16.9 §§	45.0 ± 20.1 §		58.2 ± 14.4		62.7 ± 14.7 §
	Placebo	62.6 ± 16.8	48.7 ± 13.5 §§	47.4 ± 13.5 §§		57.1 ± 15.6		59.0 ± 13.9
Concentration	SAGAT	54.7 ± 19.4	40.3 ± 17.4 §§	43.8 ± 21.9		55.0 ± 15.3		59.6 ± 16.1
	Placebo	61.4 ± 17.4	46.6 ± 12.8 §§	45.2 ± 13.9 §§		58.9 ± 16.7		61.7 ± 16.2
(D)								
		1 h after Load	2 h after Load	1 h after Recovery		2 h after Recovery		
ΔTension	SAGAT	6.9 ± 8.9	6.6 ± 13.4	−0.5 ± 10.0		−5.5 ± 10.5		
	Placebo	5.9 ± 10.4	9.5 ± 11.1	0.2 ± 11.6		−3.6 ± 11.3		
ΔSleepiness	SAGAT	15.1 ± 14.8	14.9 ± 16.8	0.2 ± 15.6		−7.1 ± 19.8		
	Placebo	12.7 ± 11.0	13.8 ± 13.0	−0.9 ± 13.4		−1.6 ± 15.0		
ΔRelaxation	SAGAT	−11.4 ± 15.5	−8.2 ± 17.4	2.9 ± 11.2		7.3 ± 14.6		
	Placebo	−13.4 ± 15.6	−14.7 ± 14.8	−0.3 ± 12.7		2.0 ± 12.6		
ΔMotivation	SAGAT	−11.4 ± 11.7	−10.1 ± 15.1	3.1 ± 10.4	] **	7.6 ± 11.1	] **	
	Placebo	−13.9 ± 13.7	−15.2 ± 14.6	−5.5 ± 9.5		−3.6 ± 9.9		
ΔConcentration	SAGAT	−14.4 ± 13.0	−10.9 ± 18.5	0.3 ± 11.5		4.9 ± 12.0		
	Placebo	−14.8 ± 13.2	−16.1 ± 13.3	−2.5 ± 8.9		± 12.6		

(A) The mean and SD of each weekly VAS recorded every morning during the weekday are shown. §§ *p* < 0.01 and § *p* < 0.05 (comparison before and after intervention). (B) The mean and SD of the changes in each VAS from before the intervention are shown. (C) The mean and SD of VAS recorded before and after transient work loading at four weeks of intervention are shown. §§ *p* < 0.01 and § *p* < 0.05 (comparisons before and after mental work loading). (D) The mean and SD of the changes in each VAS from before mental work loading at four weeks of intervention are shown. ** *p* < 0.01 (comparison between groups).

**Table 5:** Cognitive function.

(A)							
		Before		4 weeks		Changes before and after intervention	
Orientation, Time management	SAGAT	306 ± 53		328 ± 42		21 ± 51	
	Placebo	304 ± 54		321 ± 47		18 ± 77	
Attention, Follow the order	SAGAT	313 ± 40		326 ± 19		13 ± 36	
	Placebo	326 ± 16		313 ± 51		−13 ± 49	
Attention, Stroop test	SAGAT	647 ± 74	] *	699 ± 65 §		51 ± 86	
	Placebo	701 ± 75		735 ± 58		25 ± 54	
Memory, Recall	SAGAT	224 ± 21		226 ± 19		3 ± 29	
	Placebo	223 ± 20		230 ± 9		8 ± 21	
Memory, Delayed recall	SAGAT	205 ± 40		224 ± 21		19 ± 46	
	Placebo	212 ± 32		221 ± 30		14 ± 46	
Executive function, Route 99	SAGAT	98 ± 70		138 ± 53 §		40 ± 70	
	Placebo	102 ± 66		125 ± 64		25 ± 87	
Spatial cognition, Just Fit	SAGAT	103 ± 60		95 ± 65	] *	−8 ± 76	
	Placebo	118 ± 70		134 ± 55		18 ± 94	
Total score	SAGAT	1897 ± 160		2036 ± 138 §§		139 ± 157	
	Placebo	1986 ± 125		2079 ± 157 §§		95 ± 147	
(B)							
		Before Load		2 h after Load		2 h after Recovery	
Orientation, Time management	SAGAT	328 ± 42		297 ± 51		339 ± 19	] *
	Placebo	321 ± 47		295 ± 73		308 ± 49	
Attention, Follow the order	SAGAT	326 ± 19		325 ± 20		330 ± 19	
	Placebo	313 ± 51		322 ± 42		325 ± 39	
Attention, Stroop test	SAGAT	699 ± 65		694 ± 90		720 ± 52	
	Placebo	735 ± 58		720 ± 52		733 ± 58	
Memory, Recall	SAGAT	226 ± 19		215 ± 32		218 ± 32	
	Placebo	230 ± 9		216 ± 32		215 ± 29	
Memory, Delayed recall	SAGAT	224 ± 21		200 ± 43		188 ± 46 §§	
	Placebo	221 ± 30		201 ± 52		193 ± 46	
Executive function, Route99	SAGAT	138 ± 53		142 ± 56		160 ± 42	
	Placebo	125 ± 64		141 ± 42		140 ± 57	
Spatial cognition, Just Fit	SAGAT	95 ± 65	] *	116 ± 58		139 ± 54 §	
	Placebo	134 ± 55		138 ± 57		122 ± 54	
Total score	SAGAT	2036 ± 138		1989 ± 162		2094 ± 158	
	Placebo	2079 ± 157		2034 ± 162		2036 ± 145	
(C)							
		2 h after Load		2 h after Recovery			
ΔOrientation, Time management	SAGAT	−30 ± 68		12 ± 45			
	Placebo	−26 ± 77		−12 ± 65			
ΔAttention, Follow the order	SAGAT	−1 ± 16		4 ± 16			
	Placebo	9 ± 61		12 ± 64			
ΔAttention, Stroop test	SAGAT	−5 ± 77		21 ± 62			
	Placebo	−15 ± 60		−2 ± 65			
ΔMemory, Recall	SAGAT	−11 ± 37		−8 ± 26			
	Placebo	−14 ± 31		−15 ± 31			
ΔMemory, Delayed recall	SAGAT	−25 ± 50		−37 ± 50			
	Placebo	−20 ± 62		−29 ± 56			
ΔExecutive function, Route99	SAGAT	4 ± 68		22 ± 57			
	Placebo	17 ± 66		15 ± 81			
ΔSpatial cognition, Just Fit	SAGAT	22 ± 78		44 ± 81	] *		
	Placebo	4 ± 73		−12 ± 87			
ΔTotal score	SAGAT	−47 ± 141		58 ± 177			
	Placebo	−45 ± 164		−43 ± 170			

(A) The mean and SD of each index for the daily cognitive function recorded before and after intervention are shown. * *p* < 0.05 (comparison between groups). §§ *p* < 0.01 and § *p* < 0.05 (comparison before and after intervention). (B) The mean and SD of each index for cognitive function recorded before and after transient work loading at four weeks of intervention are shown. * *p* < 0.05 (comparison between groups). §§ *p* < 0.01 and § *p* < 0.05 (comparisons before and after mental work loading). (C) The mean and SD of changes in each index from before mental work loading at four weeks of intervention are shown. * *p* < 0.05 (comparison between groups).

## Data Availability

The original contributions presented in the study are included in the article/Supplementary Material; further inquiries can be directed to the corresponding author.

## Supplementary Materials

The following supporting information can be downloaded at https://www.mdpi.com/article/10.3390/nu14112357/s1: Table S1: Autonomic nervous function (other than LF, HF). (A) The mean and SD of each index for ANF recorded before and after intervention are shown. (B) The mean and SD of each index for ANF recorded before and after transient work loading at four weeks of intervention are shown. (C) The means and SD of changes in each index from before mental work loading at four weeks of intervention are shown. * *p* < 0.05 (comparison between groups). Table S2: Scores of POMS2. The mean and SD of the T-scores of each index in the POMS2 questionnaires are shown. §§ *p* < 0.01 and § *p* < 0.05 (comparisons before and after intervention). AH: Anger/Hostility, CB: Confusion/Bewilderment, DD: Depression/Dejection, FI: Fatigue/Inertia, TA: Tension/Anxiety, VA: Vigor/Activity, F: Friendliness, and TMD: Total Mood Disturbance. Table S3: Chalder fatigue scale. (A) The mean and SD of the observed value of the total score are shown. §§ *p* < 0.01 (comparison before and after intervention). (B) The means and SD of changes in total score from before intervention are shown. Table S4: Blood marker. (A) The mean and SD of each blood marker index measured before and after intervention are shown. ** *p* < 0.01 (comparison between groups). §§ *p* < 0.01 and § *p* < 0.05 (comparison before and after intervention). (B) The mean and SD of each index of blood markers measured before and after transient work loading at four weeks of intervention are shown. * *p* < 0.05 (comparison between groups). §§ *p* < 0.01 and § *p* < 0.05 (comparison before and after mental work loading). (C) The means and SD of changes in each index from before mental work loading at four weeks of intervention are shown. Table S5: Scores of ATMT. The mean and SD of the results of the ATMT (ABC task) recorded before and after intervention are shown. * *p* < 0.05 (comparison between groups). Table S6: Hematological examination before and after intervention. The mean and SD of the observed value of total score in FAS are shown. * *p* < 0.05 (comparison between groups). §§ *p* < 0.01 and § *p* < 0.05 (comparison before and after intervention).
